# Rapid response and learning for later: establishing high quality information networks and evaluation frameworks for the National Ambulance Service response to COVID-19 – the ENCORE COVID Project Protocol

**DOI:** 10.12688/hrbopenres.13149.2

**Published:** 2021-01-07

**Authors:** Siobhán Masterson, Eithne Heffernan, Dylan Keegan, Bridget Clarke, Conor Deasy, Cathal O'Donnell, Philip Crowley, Roisin Breen, Maureen E Kelly, Andrew W Murphy

**Affiliations:** 1 Discipline of General Practice, National University of Ireland, Galway, Galway, Ireland; 2 National Ambulance Service, Health Service Executive, Dublin, Ireland; 3 Emergency Department, Cork University Hospital, Cork, Ireland; 4 National Quality Improvement Team, Health Service Executive, Dublin, Ireland; 5 HRB Primary Care Clinical Trials Network Ireland, National University of Ireland, Galway, Galway, Ireland

**Keywords:** Emergency Medical Services, Information Dissemination, Health Care Evaluation Mechanisms, Coronavirus, COVID-19, Patient Care Planning

## Abstract

**Background: **The National Ambulance Service (NAS) is at the forefront of Ireland’s response to the COVID-19 pandemic. As directed in Ireland’s National Action Plan, NAS significantly expanded prehospital services, including provision of a novel COVID-19 testing service. Additionally, other health services rely on NAS’s capacity to assess, transport and/or treat COVID-19 patients. In a climate of innovation and adaptation, NAS needs to learn from international ambulance services and share experience. Evaluation of the NAS response to COVID-19 is required to facilitate evidence-based planning for subsequent waves or future pandemics, and to identify innovative practice for mainstreaming into routine service provision.

**Aims: **This project aims to test the utility of novel information networks and develop a tool that is tailored to evaluating pandemic-imposed change in an emergency medical service.

**Methods**: The first aim will be to introduce and measure the impact of ambulance-specific research and information updates for NAS. Secondly, the usefulness to members of an international network of senior ambulance and research personnel (‘AMBULANCE+COVID19’ network), and the clarity and feasibility of a short-survey instrument, the Emergency Medical Services Five Question Survey (EMS-5QS), will be assessed. Finally, an evaluation framework for assessing pandemic-imposed change will be developed to enable NAS determine innovations: (1) for reactivation in another wave or new pandemic; (2) to be sustained as part of routine service. The framework will be developed in collaboration with NAS and the National Quality Improvement Team. The Research Team includes expertise from academia, ambulance services and the National Public Health Emergency Team.

**Conclusions: **This project will facilitate the prompt introduction of information sharing processes to an emergency medical service and assess the impact of those processes. By developing a process for evaluating pandemic-imposed change in NAS, this project will add to the toolbox for future pandemic planning in emergency medical services internationally.

## Introduction

On March 11 ^th^ 2020, the World Health Organization declared the COVID-19 outbreak a pandemic ^1^. COVID-19 is an infectious disease caused by a novel coronavirus, severe acute respiratory syndrome coronavirus-2 (SARS-CoV-2), which was first identified in December 2019 ^2^. To date, there have been over 13 million confirmed cases of COVID-19 globally, including more than 580,000 deaths ^3^. In Ireland, there have been over 25,000 confirmed cases of COVID-19, including more than 1,700 deaths, to date ^4^.

The COVID-19 pandemic has impacted international emergency medical services (EMS) in different ways. Data from the US suggests that while emergency department presentations decreased for conditions such as stroke and myocardial infarction, the number of EMS attendances to deceased patients may have trebled ^5, 6^. Incidence of out-of-hospital cardiac arrest increased in Italy, New York and Paris, with significantly poorer patient outcomes also reported ^7– 9^. The nature of care provided by ambulance services changed, with efforts being made to reduce unnecessary transport to hospital ^10^, and reports of more severely ill patients refusing transport to hospital ^11^. As for all health services, ambulance personnel have had to deal with increased infection control procedures ^12^, evolving guidelines ^13, 14^, and the potential impact of the rapidly changing situation on staff wellbeing ^15^.

The National Ambulance Service (NAS) has been at the forefront of the Irish health service response to the COVID-19 pandemic. NAS is the statutory pre-hospital emergency and immediate care service provider for the Irish State. They work closely with Dublin Fire Brigade, the Irish Air Corps, the Irish Coast Guard, Community First Responder schemes, and the Northern Ireland Ambulance Service. Furthermore, private and not-for-profit providers play an important role in transporting patients requiring access to health care services and in supporting a range of public events. Intermediate care is provided by Emergency Medical Technicians, whilst prehospital emergency care is provided by paramedics and advanced paramedics. The National Emergency Operations Centre is staffed by emergency call takers, emergency medical dispatchers, control supervisors and control managers. Medical oversight is provided by a Medical Director, Deputy Medical Director and Area Medical Advisors. In terms of regulation, NAS adheres to standards developed by the independent Pre-Hospital Emergency Care Council ^16^.

As directed in the National Action Plan, NAS significantly expanded prehospital care delivery through the introduction of an entirely novel home and community COVID-19 testing service ^17^. Provision of home and community testing is a noteworthy departure for NAS. This is because prior to the pandemic, non-conveyance of patients was not an option for the vast majority of NAS practitioners unless the patient expressly refused to travel and was deemed to have the mental capacity to refuse transport ^*^. The triaging and clinical advice capacity of NAS’s National Emergency Operations Centre (NEOC) was enhanced through the formation of a multidisciplinary clinical team including a variety of nursing specialisms, general practitioners, public health doctors and Irish Defence Forces personnel. In addition, many other actions outlined the National Action Plan ^17^, such as ensuring the provision of essential patient transport to maintain healthcare access, accelerating appropriate discharge of patients to appropriate facilities or with homecare support, and enhancing paramedic led mobile medical services, have direct implications for NAS, particularly as COVID-19 patients who are moved at any point during their care will require assessment, transport and/or treatment by NAS.

In response to COVID-19, NAS provided (and continues to provide) innovative new services, adapted to mitigate impact on emergency and routine care provision and redesigned decision supports. These radical changes were achieved in unprecedented timeframes. During the pandemic, the service provided by NAS has been guided by national and internally developed guidelines, policies and directives. However, the need to learn from the actions and experiences of ambulance services worldwide is acute. It is equally important that NAS innovation and experience is shared internationally. Furthermore, the rapid pace at which these changes have been implemented, in addition to the considerable pressure placed on the ambulance service during the pandemic, have to date impeded NAS’ capacity to adequately evaluate this change.

In responding to COVID-19, NAS needs reliable, up-to-date information on scientific and organisational ambulance-related developments to assist decision making, as well as the means of swiftly communicating and collaborating with other ambulance services regarding changes to procedures and practices. In addition, a method of evaluation that can be applied to innovative and adaptive practices is needed so that planning for subsequent waves of COVID-19 or indeed another pandemic is built on evidence. Evaluation is also critical to identify aspects of innovative practice that should be mainstreamed into routine EMS service provision, and/or shared with other health services.

The aims of this research are to:

i) Establish and evaluate the impact of an information provision process for NAS staff, delivering weekly Covid-19 updates from reputable, peer–reviewed and evidence based national and international sources, communicated in a variety of ways (e.g. including print and podcasts).
ii) Develop an evaluation framework to enable NAS determine innovations: (1) for reactivation in a subsequent wave or new pandemic; (2) to be sustained as part of routine service.

This project will also provide and assess an information network and data gathering tool that will help NAS to meet these requirements and to ensure that new learning from the NAS experience is shared with EMS services internationally. At a national level, this research will impact a national healthcare system by ensuring rapid access to high quality information for all NAS staff. At an international level, this project aims to support rapid preparation for subsequent waves of COVID-19 or pandemics by creating an evaluation framework that can be used to assess the impact of service innovation as a result of the current pandemic.

## Protocol

### Research design and methodological approach

This research design for this study consists of three discrete work packages. All three work packages will be carried out simultaneously by the Research Team in collaboration with colleagues from NAS and the Health Service Executive (HSE) Quality Improvement Directorate. This research will have a mixed methods approach including systematic literature and media searching, formal literature review, social media data analysis, quantitative surveys and qualitative data analysis of semi-structured interviews. The output from all three work packages will result in the provision, development and assessment of pragmatic tools for communication and evaluation that are based on systematic review of published evidence and mixed methods evaluation of users’ experience of the utility of these tools.

***Work package 1 – Produce ambulance-specific research and information updates.*** Building on existing/ongoing work, an ‘International Update’ of COVID-19-related information from peer-reviewed sources, news and social media, ambulance service websites and podcasts will be disseminated via the NAS Medical Director to NAS staff on a weekly basis. Identification and qualitative assessment of information will be performed by the Update Editorial Board. The Editorial Board consists of researchers from NUI Galway who have training and experience in literature search techniques, as well as experience in prehospital research. The Editorial Board also includes the NAS Clinical Development Manager who is a practicing Advanced Paramedic. In order to take advantage of project synergies, the Editorial Board will be supported and advised by a lead member of the collaborative project “COVID19: Evidence for Irish general practice” (author-MK). This collaborative project was established between the Irish College of General Practitioners (ICGP), Association of University Departments of General Practice in Ireland (AUDGPI) and the HRB Primary Care Clinical Trials Network Ireland to support GPs during the COVID crisis by providing a question and expert answer service through a password protected members’ COVID19 hub of the ICGP website. In turn, a member of the Update Editorial Board will serve as a member of the expert panel for the “COVID19: Evidence for Irish general practice” project. Work package 1 will also include an investigation of the utility of social media analysis software in reviewing social media information for inclusion in weekly Updates. Throughout the project duration, interim quantitative evaluation of the Update will be carried out by the Editorial Board. In particular, feedback on the utility and usability of the Update will be obtained from members of its target audience within NAS. A final survey will be developed during the course of the research to evaluate the knowledge transfer impact of the Updates for NAS managers and staff and to assess whether this form of knowledge mobilisation should be sustained within NAS in the longer term. This survey will be underpinned by Ward *et al.*’s conceptualisation of the knowledge transfer process. Survey questions will be categorised to address each of the following components of the knowledge transfer process: problem identification and communication, knowledge development and selection, analysis of context, knowledge transfer activities, and knowledge utilisation ^18^. The use of Ward *et al.*’s conceptualisation in survey question design will provide new knowledge about how this type of information dissemination can impact knowledge transfer within an EMS organisation, particularly in a pandemic environment.

***Work package 2 – Establish an international information network and test an evidence sharing tool for senior ambulance personnel.*** As part of the research, an international network called ‘AMBULANCE+COVID19’ will be established to connect senior ambulance service managers and academics from different countries so that they can exchange information on their COVID-19 responses, practices, and insights. Research team members have contacts within ambulance services and prehospital research departments that include senior ambulance management and research personnel and will invite international colleagues to participate in the ‘AMBULANCE+COVID19’ network. AMBULANCE+COVID19 participants will be enabled to communicate directly with each other in a mode of their choice (e.g. group chat, group email) for synergy/rapid sharing of information. Research team participants will send their international network colleagues an individual invitation via email to join the AMBULANCE+COVID19 network. For the purposes of personal data protection, each Research Team member will deal with their own contacts until permission to share contact details with the wider Research Team is confirmed. If they wish to participate, international colleagues will be asked to confirm this by digitally signing a consent form. Invitees’ contact details will not be shared with the wider Research Team or AMBULANCE+COVID19 network until digital consent has been received. The network will be organised and facilitated by the Research Team, such as by recruiting participants and collating responses to queries, in order to ensure that participants remain engaged throughout and to minimise participant burden.

Under the research priority ‘Clinical Care and Health Systems’, the WHO have set this milestone:

“Establish and test pathways for dynamic flow to enable rapid sharing of evidence.” (pg.93) ^19^

To contribute to achieving this milestone, the Research Team will develop a qualitative instrument – the Emergency Medical Services Five Question Survey (EMS-5QS). The aim of the online survey will be to allow the participants to share information in a structured format on their organisation’s approach on specific aspects of service provision during the pandemic. For each survey round, one ‘AMBULANCE+COVID-19’ participant will nominate a topic for which they would like to hear about the experience and practice of other ambulance services. The participant will formulate up to five simple questions on the topic. The number of questions is limited to five to ensure focus on important aspects of the topic. While the actual topics have not been decided, the type of questions asked will strictly apply to the organisational level approach to the topic. The Research Team will advise on question formulation and will ensure that the question relates to the organisational approach and not to descriptions of individual case management or staff actions. For example, a question might be “To what type of emergency calls do your staff routinely wear Personal Protective Equipment (PPE)?”, rather than “please give a case review example where your staff wore PPE”. The Research Team will distribute questions, collate the results and share with the ‘AMBULANCE+COVID-19’ participants. Each survey will be live for five days, after which results will be shared within five days. Multiple surveys covering a range of topics will be run over the course of the research, approximately every four weeks.

It is recognised that ambulance networks that discuss COVID-19-related activity are already in existence. However, the ‘AMBULANCE+COVID19’ network differs from existing networks in several ways. Firstly, the ‘AMBULANCE+COVID19’ network will facilitate a way of communicating that is entirely subject focused and open to a broad membership of people with a managerial or research responsibility in ambulance services internationally. Additionally, through the inclusion of the EMS-5QS as an evidence sharing tool, the ‘AMBULANCE+COVID19’ network will provide a mode of communication that is systematised, standardised and rapid.

An assessment, via electronic survey, of the impact the AMBULANCE+COVID19 network on knowledge acquisition, sharing and collaboration will be carried out with network participants near the end of the study period. The survey will also assess the utility of the EMS-5QS as a structured data gathering tool for sharing information. The validity of the EMS-5QS will be considered from the perspective of: clarity i.e. were participants clear on the purpose of the tool; feasibility i.e. did participants engage in its use; and utility i.e. did participants find it to be any advantage over sharing information in a more informal format?

***Work package 3 – Create an evaluation framework to determine innovations: (1) for reactivation in the event of a new wave of COVID-19 or a new pandemic; (2) to be sustained as part of routine NAS service provision.*** Firstly, a rapid review of the literature will be carried out to identify methodologies that have been developed to evaluate health service delivery change due to pandemics or other similar medical emergencies ^20^. Literature review will be carried out in collaboration with a HSE expert librarian and using the Covidence systematic review management tool through the HSE National Health Library's Covidence account ^21, 22^. The inclusion criteria for the search will be specified according to the domains of the SPIDER search strategy tool: Sample, Phenomena of Interest, Design, Evaluation, and Research type ^23^. Data sources will include the academic databases, such as PubMed, Cochrane Central Register of Controlled Trials (CENTRAL), Embase, CINAHL, and PsycINFO. Information screening will be performed by one member of the Research Team. In order to reduce the risk of selection bias, random sample verification will be carried out by a second Research Team member. Disagreements will be settled primarily by consensus or by third-party where necessary. Quality assessment of the results will be followed by a narrative synthesis. The narrative synthesis will summarise the implications of the results found and either make recommendations for the use or modification of an existing evaluative tool or the construction of a novel tool for use in ambulance services.

Secondly, the framework will be developed with guidance and collaboration from the HSE National Quality Improvement Team, including agreement on the data collection tool and the outcomes that will determine innovation success. The acceptability and feasibility of the agreed evaluation tool will be tested with the staff who worked in the ‘COVID-19 room’ in National Emergency Operations Centre (NEOC) – a dedicated centre for dealing with requests and arranging for COVID-19 home and residential testing by NAS. As the aim of this research is to develop the evaluation tool, the tool content or method of administration is not known at the outset of the project. The evaluation tool will be assessed for (1) content validity i.e. degree to which the tool adequately measures the domains of evaluation included in the evaluation framework and (2) structural validity i.e. the degree to which the tool enables measurement of change from the individual and organisational levels. Reliability of the tool will also be assessed by checking that questions are understood and answered by respondents in a similar way. As a number of Research Team members have managerial positions in NAS, in order to avoid a risk of hierarchical bias in recruitment, the NAS human resources department will email an invitation to participate to COVID Room staff members. The identity and contact details of staff members will only be shared with the Research Team when the human resources department has received a digitally signed consent form from the staff member that they are willing to participate.

### Ethics

Ethical approval has been granted by the COVID-19 National Ethics Research Committee (20-NREC-COV-025). The research will be conducted in accordance with the General Data Protection Regulation.

### Dissemination

For work package 1, weekly updates will be shared via email with NAS leadership who will disseminate updates to NAS staff. Knowledge exchange will be further facilitated through collaboration with the “COVID19: Evidence for Irish general practice” project (as described above). Work package 2 involves the development of a survey tool (i.e. EMS-5QS) that will be designed to facilitate rapid and systematised knowledge exchange among international ambulance services. The work package is specifically designed to take advantage of the existing international networks of Research Team members. Networks members will receive regular survey results. Dissemination of work package 3 will be developed with the guidance of the HSE Quality Improvement Division. As a member of the Research Team is part of the National Public Health Emergency Team (NPHET), there will be a direct link for sharing outputs. The Research Team intend to make the EMS-5QS and evaluation frameworks available to international colleagues. Project updates will be provided on the NUI Galway website and a dedicated Twitter account. Findings will be presented face-to-face or online to NAS. In the mid/longer term, findings will be disseminated via journals (open access) and attendance at (online) conferences. The project will also be registered with the World Health Organisation database of research on COVID-19.

### Study status

The study commenced on 11 ^th^ May 2020. To date (September 2020), international updates of COVID-19-related information has been disseminated on a weekly basis to NAS staff and managers, and initial evaluation of the updates has been carried out with a sample of recipients. As a result, the Editorial Team will make amendments based on the feedback received. Review of social media analysis tools is also underway. The AMBULANCE+COVID19 network has been launched and the first round of the EMS-5QS survey is currently being assessed. Finally, the protocol for the rapid literature review described for work package 3 has been published on PROSPERO ( CRD42020198657), the international prospective register of systematic reviews which is curated by the National Institute of Health Research in the United Kingdom ^20^.

## Conclusions

The primary aim of this project is to provide information networks and evaluation methodologies that help the NAS to meet their information needs and to learn from pandemic-imposed change. During the COVID-19 pandemic, Irish health services – with the guidance of the Health Protection Surveillance Centre and the Health Information and Quality Authority, has excelled in the provision of high quality and consistent guidelines and advice to health service providers. However, the proliferation of information and publications relating to COVID-19 is exceptional ^24^. Reliable ways to filter, receive and share high quality information specifically relating to prehospital emergency care are needed by ambulance managers and staff. Coupled with this, ambulance services are facing unprecedented service change at a pace that has not been previously experienced. Despite changes in the type of services provided, the NAS is, at heart, an emergency service and culturally well equipped to react to unexpected change. However, without effective evaluation strategies, learning from this unique period in emergency health care provision will be limited. This project will provide, test, and systematically evaluate information and evaluation frameworks for the Irish ambulance services, and ensure that learning is shared with the wider prehospital ambulance and research community.

Key strengths of this project include:

The capacity for immediate impact through weekly publication and dissemination of weekly ‘International Updates’ to NAS staff and managers.

Synergy with existing information sharing projects, particularly the ““COVID19: Evidence for Irish general practice” with the accompanying opportunity for application of similar evaluation methodologies and subsequent comparison.

Building on existing networks, this project can use the ‘opportunity of a crisis’ to strengthen the practice of networking in the prehospital community.

Production of a review of the literature on evaluation of pandemic-imposed change and assurance that an evaluation framework that is suited to ambulance services is developed.

Finally, there is room for increased ambulance service involvement in research in Ireland. This project presents an important opportunity to help build a culture of research around ambulance service innovation and evaluation.

## Data availability

No data are associated with this article.

## Notes

* Until the pandemic, Community Paramedics were the only category of NAS Practitioner that could attend and treat a patient without conveyance to hospital. This specialist category of practitioner is in the process of being introduced to NAS and as a result, at the beginning of 2020, only three Community Paramedics were in practice in the Republic of Ireland.
