# Supplementary material for: Rapid response and learning for later: establishing high quality information networks and evaluation frameworks for the National Ambulance Service response to COVID-19 – the ENCORE COVID Project Protocol
Source: HRB Open Res. 2021 Jan 7;3:68. Originally published 2020 Sep 22. [Version 2] doi: 10.12688/hrbopenres.13149.2 (PMC7836028; doi:10.12688/hrbopenres.13149.2)
Supplement: Peer review history

# Peer review: Reviewer response for version 2

**DOI:** 10.21956/hrbopenres.14358.r28622

**Published:** 2021-01-21

**Authors:** Julia Williams

**Affiliations:** 1 University of Hertfordshire, Hatfield, UK

Thank you for your responses to my previous comments. You have answered the questions I had and I have no further comments to make. 

 I just want to wish the team success with the study which I believe is underway and I look forward to reading about your study's findings in the future.

Is the study design appropriate for the research question?

Partly

Is the rationale for, and objectives of, the study clearly described?

Partly

Are sufficient details of the methods provided to allow replication by others?

Partly

Are the datasets clearly presented in a useable and accessible format?

Not applicable

Reviewer Expertise:

Ambulance service research, Prehospital emergency medicine research, Paramedic education.

I confirm that I have read this submission and believe that I have an appropriate level of expertise to confirm that it is of an acceptable scientific standard.

# Peer review: Reviewer response for version 1

**DOI:** 10.21956/hrbopenres.14265.r27988

**Published:** 2020-11-30

**Authors:** Julia Williams

**Affiliations:** 1 University of Hertfordshire, Hatfield, UK

Overall this is an interesting piece of work and clearly has value in terms of the ultimate outcomes of this work. To strengthen the publication, it would be helpful if the authors just clarify some areas in order to clearly demonstrate whether this is research or service development/improvement.

 It is my opinion that the aims in the abstract and core text of the introduction do not immediately illustrate that this is research.

 WP1 seems to be the collection and dissemination of publicly accessible material. It then appears that there will be some form of evaluation designed to assess how effective these materials have been and what the staff's experiences are of having used them. Clearly this can be packaged into a variety of constructs but one of them could most certainly be research. You just need to make this absolutely clear for the reader. One area that must be clarified is when you outline that you intend to evaluate the impact of this new system by developing a framework informed by Ward *et al'*s work, you should clarify for the readers how you will actually achieve this. I think this is especially important with regard to the final three components: analysis of context, knowledge transfer activities and knowledge utilisation.

 WP2 would benefit from more focused description. Is the setting up of the network part of a research intervention? Your focus is clearly to 'Test pathways for dynamic flow to enable rapid sharing of evidence'. One aspect that is not clear to me, apologies for that, but is the EMS-5QS a vehicle for sharing information, or are you collecting data about people's experiences? Whatever the answer we need to know how you are going to test this tool for validity. So more information is needed to improve the clarity of the activities in this work package.

 In WP3, the approach to rapid review is clear. I realise that the evaluation tool will be developed as part of this study so we do not know what it comprises at this point in time, but it would be helpful to explain how you intend to confirm that your evaluation tool is valid as whilst you mention testing it for feasibility there is no other detail.

 In conclusion, you outline that you will be creating and developing resources but, at times it is not clear how you will achieve this. I hope this is helpful.

 Could I also just make a note that when selecting 'Approved with Reservations', I received a text box saying that a sentence will be added to my comments saying I have 'significant' concerns. I do not have 'significant' concerns, but there are some areas that need improved clarity to to demonstrate that this is fundamentally a research project rather than a service improvement initiative - although at times the two are in alignment.

Is the study design appropriate for the research question?

Partly

Is the rationale for, and objectives of, the study clearly described?

Partly

Are sufficient details of the methods provided to allow replication by others?

Partly

Are the datasets clearly presented in a useable and accessible format?

Not applicable

Reviewer Expertise:

Ambulance service research, Prehospital emergency medicine research, Paramedic education.

I confirm that I have read this submission and believe that I have an appropriate level of expertise to confirm that it is of an acceptable scientific standard, however I have significant reservations, as outlined above.

# Response

**Published:** 2020-12-22

**Authors:** Siobhan Masterson

**Affiliations:** National University of Ireland Galway, Ireland

Thank you for the thoroughly considered review of our project protocol. We agree with your observations that there is a need to outline more clearly that this is a research project, albeit with a substantial quality/service improvement dimension. We have provided responses to your observations below and have amended the protocol text where we felt it was appropriate. We hope that we have sufficiently addressed the issues you have raised and hope that the manuscript has been improved as a result. 

 info_outline

*Overall this is an interesting piece of work and clearly has value in terms of the ultimate outcomes of this work. To strengthen the publication, it would be helpful if the authors just clarify some areas in order to clearly demonstrate whether this is research or service development/improvement.*

- Thank you. Please see below for responses to each observation made.

*It is my opinion that the aims in the abstract and core text of the introduction do not immediately illustrate that this is research.*

- We have reviewed the abstract and core text of the introduction and agree with your opinion that there is a need to more clearly illustrate that this is a research project. We have rewritten the abstract so that it focuses not only on the information processes that will be introduced, but also on the methods of assessment and evaluation tool development that will be used throughout the project.

*WP1 seems to be the collection and dissemination of publicly accessible material. It then appears that there will be some form of evaluation designed to assess how effective these materials have been and what the staff's experiences are of having used them. Clearly this can be packaged into a variety of constructs but one of them could most certainly be research. You just need to make this absolutely clear for the reader. One area that must be clarified is when you outline that you intend to evaluate the impact of this new system by developing a framework informed by Ward et al's work, you should clarify for the readers how you will actually achieve this. I think this is especially important with regard to the final three components: analysis of context, knowledge transfer activities and knowledge utilisation.*

- We agree that it is important to more clearly describe how WP1 fits into a research construct. Our research aim is to understand the knowledge transfer impact of this type of information dissemination for our managers and staff. We believe that a survey will be the most appropriate method of assessing the impact of this type of information dissemination. The survey method will also enable us to assess the appropriateness of continuing information dissemination beyond the end of the research project. Text has been added to the description of WP1 to better describe how Ward et al.’s conceptualisation of knowledge transfer activities will be used to design the final survey in WP1, and how this will allow us to generate new knowledge about the effectiveness of this method of information dissemination, particularly in a pandemic setting.

*WP2 would benefit from more focused description. Is the setting up of the network part of a research intervention? Your focus is clearly to 'Test pathways for dynamic flow to enable rapid sharing of evidence'. One aspect that is not clear to me, apologies for that, but is the EMS-5QS a vehicle for sharing information, or are you collecting data about people's experiences? Whatever the answer we need to know how you are going to test this tool for validity. So more information is needed to improve the clarity of the activities in this work package.*

- Setting up the network is indeed part of the research intervention. The primary purpose of this intervention is to assess whether the network can effectively facilitate knowledge acquisition and sharing for the participants, and also to assess if systematising knowledge sharing using the EMS-5QS is of benefit to participants. Your question in relation to the purpose of the EMS-5QS is very reasonable. The EMS-5QS is a data gathering tool only and validity will be assessed in terms of clarity, feasibility and utility. It has now been clarified at the start of the description of WP2 that the AMBULANCE+COVID19 network is a research intervention, and a short paragraph has been added to the end of this section to describe the evaluation process that will be used.

*In WP3, the approach to rapid review is clear. I realise that the evaluation tool will be developed as part of this study so we do not know what it comprises at this point in time, but it would be helpful to explain how you intend to confirm that your evaluation tool is valid as whilst you mention testing it for feasibility there is no other detail.*

- Based on the findings from the rapid review and in consultation with the HSE National Quality Improvement Team, an evaluation framework for evaluating pandemic imposed change will be decided and an evaluation tool created. The acceptability of the tool as a method of evaluation for staff will be assessed, as will feasibility for use beyond the study period. Tool validity will be considered under the dimensions of content and structure. Text has been added to the description of WP3 which further outlines this plan for validity assessment.

*In conclusion, you outline that you will be creating and developing resources but, at times it is not clear how you will achieve this. I hope this is helpful.*

- Thank you. Your review was most helpful and encouraged us to ensure that the research aims of our project are more clearly described in this protocol.

*Could I also just make a note that when selecting 'Approved with Reservations', I received a text box saying that a sentence will be added to my comments saying I have 'significant' concerns. I do not have 'significant' concerns, but there are some areas that need improved clarity to demonstrate that this is fundamentally a research project rather than a service improvement initiative - although at times the two are in alignment.*

- Thank you, understood.

# Peer review: Reviewer response for version 1

**DOI:** 10.21956/hrbopenres.14265.r28340

**Published:** 2020-11-25

**Authors:** Paul Jennings

**Affiliations:** 1 Department of Paramedicine, Monash University, Melbourne, Australia

This is an important project and the authors have provided a very well written project protocol which will inform the EMS and Emergency communities of work which is currently underway.

 The rationale and aims have been clearly stated. The proposed study design is appropriate, and enlists several qualitative and quantitative methodologies across 3 ‘work packages’. Given the role of some of the investigators in the study (senior NAS managers) the investigators intend to assign recruitment of informants / participants to an independent department to avoid selection issues associated with perceived power inequalities and coercion which is prudent. The methods are sufficiently detailed to allow replication in all areas except the literature review, which would only benefit from inclusion of the proposed search strategies. No doubt this will be covered in much more detail in the literature review manuscript arising from this body of work.

 Relying on mixed methods methodologies, the authors propose to undertake three essentially distinct work packages; each of which will provide useful information to inform the current dearth of EMS focused knowledge, the utility of which will support National and International efforts in managing this (and likely future) pandemics.

 I understand from the 'Study Status' section that work is currently underway, and I wish the authors success and look forward to reviewing the output arising from the three bodies of work.

Is the study design appropriate for the research question?

Yes

Is the rationale for, and objectives of, the study clearly described?

Yes

Are sufficient details of the methods provided to allow replication by others?

Yes

Are the datasets clearly presented in a useable and accessible format?

Not applicable

Reviewer Expertise:

Prehospital clinical trials, observational studies and systematic reviews.

I confirm that I have read this submission and believe that I have an appropriate level of expertise to confirm that it is of an acceptable scientific standard.

# Response

**Published:** 2020-12-22

**Authors:** Siobhan Masterson

**Affiliations:** National University of Ireland Galway, Ireland

Thank you for your review of our protocol. We are very pleased that you were satisfied with the protocol and look forward to sharing the results of our project.
